# Study of the Antioxidative Effects of *Bombyx mori* Silk Sericin in Cultures of Murine Retinal Photoreceptor Cells

**DOI:** 10.3390/molecules27144635

**Published:** 2022-07-20

**Authors:** Shuko Suzuki, Onur Sakiragaoglu, Traian V. Chirila

**Affiliations:** 1 Queensland Eye Institute, South Brisbane, QLD 4101, Australia; shuko.suzuki@qei.org.au (S.S.); onur.sakiragaoglu@qei.org.au (O.S.); 2 School of Chemistry & Physics, Queensland University of Technology, Brisbane, QLD 4001, Australia; 3 Australian Institute of Bioengineering & Nanotechnology (AIBN), University of Queensland, St Lucia, QLD 4072, Australia; 4 Faculty of Medicine, University of Queensland, Herston, QLD 4006, Australia; 5 School of Molecular Sciences, University of Western Australia, Crawley, WA 6009, Australia; 6 Faculty of Medicine, George E. Palade University of Medicine, Pharmacy, Science and Technology, 540139 Târgu Mures, Romania

**Keywords:** silk sericin, oxidative stress, antioxidants, hydrogen peroxide, retinal photoreceptor cells

## Abstract

The availability of natural substances able to fulfill the role of antioxidants in a physiologic environment is important for the development of therapies against diseases associated with excessive production of reactive oxygen species and ensuing oxidative stress. Antioxidant properties have been reported episodically for sericin, a proteinaceous constituent of the silk thread in the cocoons generated by the larvae of the *Lepidoptera* order. We investigated the sericin fractions isolated from the cocoons spun by the domesticated (*Bombyx mori*) silkworm. Three fractions were isolated and evaluated, including two peptidoid fractions, the crude sericin and the purified (dialyzed) sericin, and the non-peptidoid methanolic extract of the crude fraction. When subjected to Trolox equivalent antioxidant capacity (TEAC) assay, the extract showed much higher antioxidant capacity as compared to the crude or purified sericin fractions. The three fractions were also evaluated in cultures of murine retinal photoreceptor cells (661 W), a cell line that is highly susceptible to oxidants and is crucially involved in the retinopathies primarily caused by oxidative stress. The extract displayed a significant dose-dependent protective effect on the cultured cells exposed to hydrogen peroxide. In identical conditions, the crude sericin showed a certain level of antioxidative activity at a higher concentration, while the purified sericin did not show any activity. We concluded that the non-peptidoid components accompanying sericin were chiefly responsible for the previously reported antioxidant capacity associated with sericin fractions, a conclusion supported by the qualitative detection of flavonoids in the extract but not in the purified sericin fraction.

## 1. Introduction

Sericin is the second major (∼25%) protein in the composition of the silk thread produced by the domesticated silkworm *Bombyx mori* and related species, accompanying fibroin (∼75%) as an adhesive coating layer. Both proteins can be readily regenerated from raw silk (either cocoons or silk yarn) in laboratory conditions. The silk cocoons of the genus *Bombyx* contain the highest amounts of sericin of all cocoons produced by the Lepidopteran larvae [1]. While silk fibroin has found many applications as a biomaterial, *B. mori* silk sericin (henceforth, BMSS) has been largely neglected on account of causing immunogenic tissue responses. However, other investigators [2,3,4] have suggested the lack of such responses. More recent studies [5,6,7,8], based on a critical examination of the extant literature or on further experiments, have proved that BMSS did not induce cytopathologic effects and also have suggested that the earlier opinions were due to insufficient documentation or poor experimental design, occasionally aggravated by instances of misinterpretation or misquotation of the reported results. Investigations of the beneficial effect of BMSS as a supplement in cell culture media [9,10,11,12,13,14,15,16,17,18,19] were carried on consistently over the past two decades leading to the current commercial availability of sericin-based supplements. In the meantime, the biocompatibility of BMSS became generally accepted, triggering increased interest in the potential applications of BMSS in tissue engineering and biomedicine [20,21,22,23,24,25,26,27,28,29,30]. Apart from sericin’s reported enzyme inhibiting and cytoprotective effects, its activity as an *antioxidant* (also termed antioxidative capacity) is another of its remarkable biological properties, which was first noticed about two decades ago [31].

Oxidative stress is commonly defined as a state of imbalance between the amount of the oxidant *reactive oxygen species* (ROS) generated by cells and the endogenous antioxidants within the cell’s physiological environment, resulting in excess production of ROS. Although frequently used, the generality of this concept has been debated [32,33,34], and the validity of the oxidative stress theory of disease has been lately challenged [35,36,37]. 

The antioxidants (either endogenous or exogenous) are substances able to delay or prevent the formation of ROS (free radicals, ions, radical-ions, and other reactive molecular species) from oxidizable substrates (proteins, lipids, carbohydrates, and nucleic acids) by any of such actions as inhibiting the formation of free radicals or scavenging them, binding to substances that may promote the formation of ROS, or involving more complex mechanisms [38,39,40,41,42]. Certain processes involving ROS are necessary for life [34,43,44]. However, most ROS are in a chemically unstable state, and they trade electrons with the surrounding matter, including the intracellular organelles, to reach a stable state. Such processes lead to oxidative stress and injury to cells, tissues or organs, especially manifested when ROS levels have escaped from under homeostatic control and become in excess. 

As recounted in some seminal reviews [40,41,42,43,44,45,46,47,48,49,50,51,52,53,54,55,56], there is a vast body of evidence showing that for organisms living in an atmosphere containing oxygen, oxidative stress is an unavoidable consequence that leads to aging and diseases. However, if antioxidants are present, they can partially control the production of ROS when the cells’ own defense mechanisms no longer can act efficaciously. Out of all our organs (besides skin), the eye is associated with the most auspicious circumstances for oxidative damage. It is relentlessly exposed to the oxygen permeating from the atmosphere and to the electromagnetic radiation provided by the sun, notwithstanding that light is essential for our vision. In the ocular tissues, the “oxidative photodegradation triangle” [57]―an analogy of the “fire triangle”―is fulfilled perhaps like nowhere else in the body, as it comprises all necessary elements, namely radiation (the “ignition source”), tissular radiation-absorbing chromophores (the “fuel”), and oxygen (as itself), an ideal synergy for generating excessive amounts of ROS, all enclosed in a relatively small organ of our body. 

BMSS has been reported as an antioxidant in preparations isolated directly from cocoons, generally followed by additional purification, and then assessing its antioxidant capacity either by a variety of chemical assays [31,58,59,60,61,62,63,64,65] or in cell cultures [61,63,65,66,67,68,69,70]. Antioxidant activity has also been demonstrated in the non-peptidoid compounds associated with sericin that were isolated by various separation techniques [60,65,71,72,73,74]. It was reported that the antioxidative capacity of isolated sericin was not affected by its chemical modification [75] nor by additional enzymatic degradation [76,77]. However, when retinal cells were cultured on solid fibroin-sericin substrates (as hydrogel films), no antioxidative protection induced by the presence of sericin was noticed [78]. BMSS has also been applied as a potential therapeutic agent (administered either by diet, topically, or by perfusion/injection) in animal pathologic models of skin damage, tumors, hyperglycemia, or myocardial infarction [60,79,80,81,82,83,84], where the observed therapeutic effects were attributed to a decline in the level of oxidative stress.

We can conclude that, amongst the two major constitutive proteins of silk, the antioxidant activity is specific to sericin, at least in *B. mori* species. However, it is still unclear whether this activity can be attributed (a) solely to sericin, with or without the accompanying peptidoid entities resulting from the hydrolytic degradation during isolation procedures; (b) solely to the non-protein substances associated with sericin (if so, this may imply that routine purification procedures for sericin may not be sufficient to remove such compounds); (c) to both previous components; or (d) it is a combined effect of a gland-secreted complex that consists of the main constituent native sericinoid polypeptides, non-sericinoid polypeptides, and non-protein organic substances. To investigate the alternative (d) above is both difficult and impractical, as it would involve the isolation of native sericin directly from the silkworm’s middle gland through an aspirating device, precisely at a stage prior to coating the fibroin filaments, and assuring that no alteration takes place during its experimental manipulation. 

Here, we report results that may contribute to further understanding of some aspects of the topic. In the present study, hydrogen peroxide was used as an oxidant agent. The antioxidant effects of sericin fractions and of its methanolic extract were evaluated employing cultures of a murine retinal photoreceptor cell line (661 W) in parallel with a routine chemical assay. This cell line had been cloned [85] from retinal tumors generated in a transgenic mouse expressing the simian virus SV 40 T-antigen under the control of human interphotoreceptor retinoid-binding protein (IRBP) promoter. The 661 W cells express cone markers, with no rod markers detectable, are sensitive to light [86], and appear to be a valuable model for the investigation of various retinopathies where oxidative stress is believed to be a major causal factor [87,88,89].

## 2. Materials and Methods

### 2.1. Materials

Silk cocoons (*B. mori*) were supplied by Tajima Shoji Co. Ltd. (Yokohama, Japan), with the pupae removed. According to the supplier, these silkworms were fed at their early stages of life with an artificial diet consisting of mulberry leaf powder, starch, and defatted soybean powder. At later stages, they were fed exclusively on fresh, natural mulberry leaves. 

The chemical reagents were all supplied by MilliporeSigma (St Louis, MO, USA), which also supplied the dialysis tubes with an MMCO of 3.5 kDa. High-purity water (Milli-Q or equivalent) was used in all procedures. The Minisart^®^-GF pre-filters (0.7 μm) and sterile Minisart^®^ High-Flow filters (0.22 μm) were supplied by Sartorius Stedim Biotech (Göttingen, Germany). All cell culture reagents and supplements were purchased from Thermo Fisher Scientific (Rockford, IL, USA), except for the fetal bovine serum (FBS) that was supplied by Cytiva (Sydney, Australia).

The 661 W murine retinal photoreceptor cell line originated in Professor Muayyad Al-Ubaidi’s laboratory at the University of Oklahoma Health Science Center (Oklahoma City, OK, USA). Dr. Krisztina Valter-Kocsi (Australian National University Medical School, Canberra, Australia) provided this line for our experiments.

### 2.2. Isolation of BMSS from Silk Cocoons

Autoclave extraction was applied according to a published protocol [90], with some modifications. In the current study, the cocoon material (unwashed, 10 g), placed in 200 mL water, was autoclaved at 121 °C for 4 h. Two different sample variants were further processed. A *purified* sericin powder (henceforth, PS) was prepared by dialysis of the solution using tubes with MMCO of 3.5 kDa in water for 2 days at constant 30 °C, with continuous stirring. Prior to dialysis, the solution was passed through a paper filter (Whatman #4), and after dialysis through successive sterile Minisart^®^ filters (0.7 and 0.22 μm). Following the last filtration step, the solution was frozen at –80 °C and concentrated to a powder in a freeze-dryer/vacuum concentrator (Alpha 1-2 LDplus, Martin Chris GmbH, Osterode, Germany). To obtain the *crude* sericin powder (henceforth, CS), the same procedure was followed but omitting the dialysis stage and post-dialysis filtration. The sericin powder samples were all stored at room temperature until further use.

### 2.3. Electrophoretic Analysis of Sericin Fractions

The molecular mass distributions in CS (i.e., non-dialyzed) and PS (i.e., dialyzed) samples were analyzed by sodium dodecyl sulfate-polyacrylamide gel electrophoresis (SDS-PAGE) employing a Novex^®^ XCell Sure Lock™ Mini-Cell system (Life Technologies, Carlsbad, CA, USA), according to a previously published protocol [90] that has been slightly modified. Thus, the aqueous solution of sericin (10 mg/mL) was mixed with both NuPAGE^®^ LDS sample buffer and NuPAGE^®^ sample reducing agent and heated at 70 °C for 10 min. A volume of 10 μL BMSS solution containing about 20 μg protein was then loaded into 1 mm thick 4–12% NuPAGE^®^ Bis-Tris gel in NuPAGE^®^ MES SDS running buffer. The gels were run at a voltage of 200 V for 35 min together with the SeeBlue^®^ Pre-stained Protein Standard. The remaining procedure was carried out as detailed previously [90]. 

### 2.4. Extraction of Non-Sericin Fraction from Crude BMSS

Freeze-dried CS powder (1.5–2 g) was placed in a screw-capped bottle with 150 mL methanol and shaken at 240 rpm for 2 days at room temperature on a shaker (Model 130 Basic, IKA, Staufen, Germany). The resulting liquid was filtered through a filter paper (Whatman #4) and then through successive Minisart^®^ filters. The solution (henceforth, CS-E) was concentrated to about 15 mL in a rotary evaporator (Rotavapor R-215, BÜCHI Labortechnik AG, Flawil, Switzerland), subsequently dried in an oven at 60 °C overnight, and then kept in a vacuum oven at 40 °C for 2–3 days under moderate vacuum. The dried CS-E was stored at 4 °C until further use. 

### 2.5. Analysis of Flavonoids in Sericin Fractions and Extract

To check the presence of flavonoids and their distribution in the sericin fractions or the extract, two chromogenic methods were employed. 

(a) The first method was based on the staining induced by the reaction of polyphenolic compounds, such as flavonoids, with 4-(dimethylamino)cinnamaldehyde (DAC) (Sigma Cat. D4506; IUPAC name: (E)-3-[4-(dimethylamino)phenyl]prop-2-enal). This reagent is to be stored in a freezer, and its solutions shall be kept in cool dark conditions. According to our protocol, 0.1 g DAC was dissolved in a pre-cooled mixture of 25 mL concentrated hydrochloric acid and 70 mL methanol. The same amounts (10 mg) of CS, PS, or CS-E were dissolved in 0.1 mL water in test tubes, and the resulting solutions were mixed each with 2 mL DAC solution and vortexed briefly. Color change was observed after keeping the test tubes on the bench for 1 h at room temperature.

(b) The visualization of flavonoids was based on the fluorescence generated by their complexation reaction with 2-aminoethyl diphenylborinate (2-APB) (Sigma Cat. D9754; IUPAC name: 2-[(diphenylboranyl)oxy]ethan-1-amine). The reagent was dissolved in water containing 0.3% dimethyl sulfoxide to achieve a concentration of 0.2% (*w*/*v*). The samples (CS, PS, and CS-E) were dissolved in water to a concentration of 5 mg/mL. Equal volumes of 2-APB and sample solutions were mixed in Eppendorf tubes and vortexed thoroughly. Volumes of 100 μL of each mixture were placed in the wells of a black 96-well plate and read on a fluorescent microplate reader (FLUOstar OPTIMA, BMG Labtech Pty Ltd., Mornington, Australia) at an excitation wavelength of 360 nm and an emission wavelength of 415–425 nm. A solution of 2-APB as such was used as a blank, and its intensity was subtracted from the sample intensities to provide the background level.

### 2.6. Protein Quantitation in Extract

The dried CS-E was dissolved in water to make up a 1 mg/mL solution and then diluted 10-fold to obtain a concentration suitable for analysis. The quantitation of proteinaceous matter was carried out using the Micro BCA protein assay (Pierce™ BCA Protein Assay Kit, Thermo Fisher Scientific, Rockford, IL, USA) and following the manufacturer’s guidelines. Briefly, sample solutions (150 µL) were mixed with the working reagent (150 µL) in a 96-well plate and incubated for 2 h at 37 °C. Absorbance was then measured at 562 nm using the microplate spectrometer AC200D (Paradigm Absorbance Detection, Beckman Coulter, Brea, CA, USA). Bovine serum albumin standard solution (included in the kit) was sequentially diluted and used to obtain a standard curve (0–200 µg/mL). Each sample was assessed in triplicate.

### 2.7. Trolox Equivalent Antioxidant Capacity (TEAC) Assay

The sericin powders, both CS and PS, and the dried CS-E were each dissolved in water to a concentration of 10 mg/mL. These solutions were assessed for their intrinsic antioxidant capacity with the TEAC assay employing a dedicated kit (Cayman Chemical, Ann Arbor, MI, USA) and following the manufacturer’s instructions. Essentially, this assay indicates the capacity of a substance to prevent the formation of the radical cation of 2,2′-azinobis(3-ethylbenzothiazoline-6-sulfonic acid) (ABTS**^+·^**) compared to the standard antioxidant 6-hydroxy-2,5,7,8-tetramethylchroman-2-carboxylic acid (a substance related to tocopherols and commonly known as Trolox). Briefly, 10 µL sample solution was mixed with metmyoglobin (10 µL) and chromogen solutions (150 µL), and a reaction was initiated by adding 40 µL hydrogen peroxide (H_2_O_2_). After incubating on a shaker for 5 min at room temperature, the absorbance was recorded at 405 nm using the microplate spectrometer AC200D. Trolox solutions of varying concentrations were used as standards for a calibration plot (from 0 to 0.33 mM), and the Trolox equivalents of antioxidant capacity were calculated per 1 mg of sample. Each sample was assessed in triplicate.

### 2.8. Cell Culture and the Effect of Oxidant on Cell Viability

Using a previously reported protocol [87], the initial culture of 661 W cells was established using T75 tissue culture flasks in Dulbecco modified Eagle’s medium (DMEM) supplemented with 10% FBS, 2 mM L-glutamine, 50 U/mL penicillin, and 50 mg/mL streptomycin. The harvested cells were seeded onto 24-well plates at a density of 25,000 cells/well and kept in an incubator for 24 h. A CO_2_ incubator Model MCO-170AICL (PHCbi, Tokyo, Japan) was used for all cell culture experiments.

The powder samples CS, PS, and CS-E were each dissolved in DMEM supplemented with 1% FBS and then filter-sterilized by passing through a sterile 0.22 μm filter. The medium in each well was replaced with 0.5 mL of the test sample solutions and incubated for 24 h, using 6 wells for each sample and DMEM/1% FBS as a control. Within each group, three wells served as controls, while the other three wells were supplemented with 1.2 mM H_2_O_2_ as a solution of 60 mM in DMEM/1% FBS (made by diluting the as-supplied H_2_O_2_ of 30% in water) and further incubated for 24 h. The medium was then removed, and all cultures were washed briefly with Hank’s balanced salt solution (HBSS).

The MTT assay was used to evaluate quantitatively the cell viability following the H_2_O_2_-induced oxidative treatment. Briefly, stock solutions of 5 mg/mL 3-(4,5-dimethylthiazol-2-yl)-2,5-diphenyltetrazolium bromide (MTT) were prepared in Dulbecco’s phosphate buffered saline, filter-sterilized, and stored at –20 °C until required. Prior to running the assay, the stock solution was diluted 10-fold with DMEM/1% FBS medium to achieve a working solution of 0.5 mg/mL. HBSS was aspirated from wells, 1 mL of MTT solution was added to each well, and the samples were all incubated for 3 h at 37 °C. After this, the MTT supernatant was aspirated and discarded, and 1 mL of a 0.04 N solution of hydrochloric acid in isopropanol was added to each well and shaken gently for 5 min. From each well, 200 μL of isopropanol solution was transferred to wells of a new 96-well plate, and the absorbance was measured at the 570-nm wavelength in an AC200D microplate spectrophotometer. The percent cell viability was then estimated against the readings for the controls taken as 100% viability.

### 2.9. Microscopic Procedure

The morphology of proliferating cells was examined and photographed by using a bright-field Nikon Eclipse^®^ TS100 microscope (Nikon, Tokyo, Japan) equipped with a Nikon Digital Sight camera and using the NIS Elements^®^ F4.00.00 software.

## 3. Results

**Figure 1:**
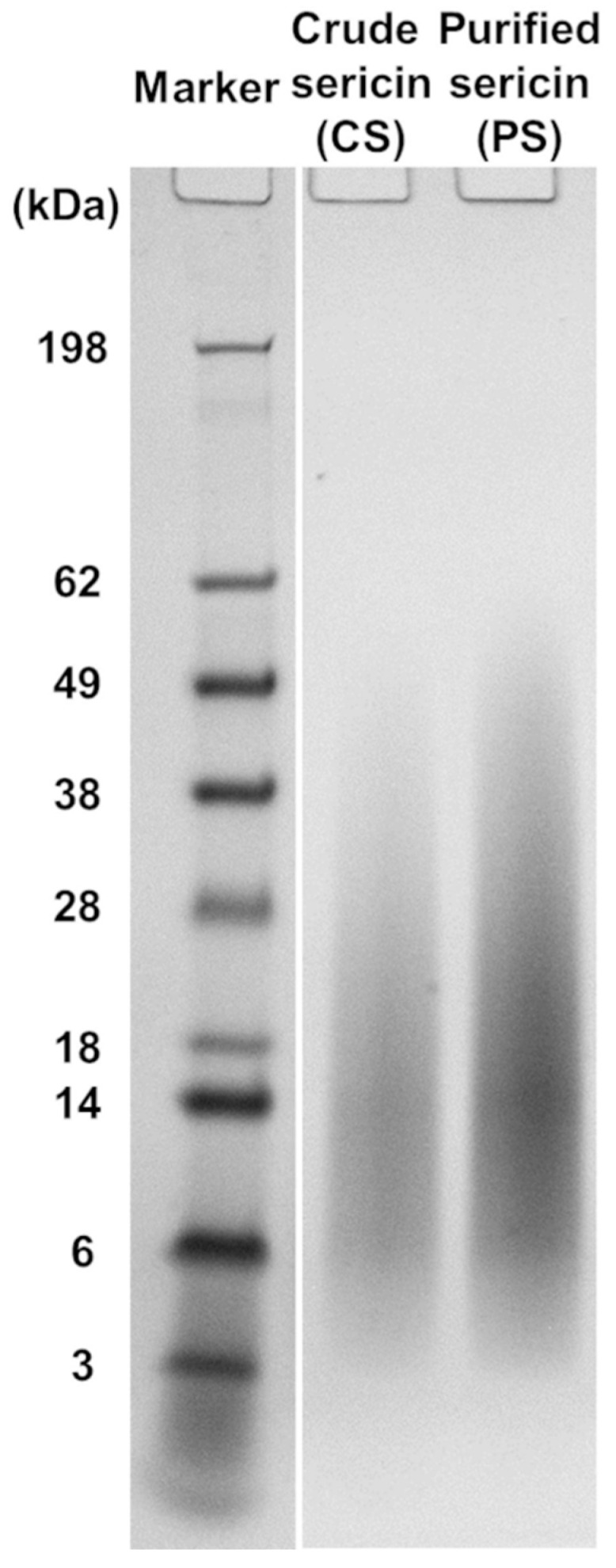
Molecular mass distribution in the sericin regenerated from *B. mori* cocoons, as determined by SDS-PAGE. The electrophoretic patterns of non-dialyzed (crude) and dialyzed (purified) products are shown.

Two BMSS samples were regenerated from silk cocoons for this study: a crude product (CS) resulting directly from autoclaving as an aqueous solution and a purified fraction (PS) that was obtained after extensive dialysis of CS, in a process that had removed most of non-peptidoid and some of peptidoid substances of low molecular mass. The yields of sericin fractions relative to the initial weight of cocoon material were determined gravimetrically and found to be 15.49% for CS and 10.28% for PS. The electrophoretic analysis of CS and PS samples resulted in similar smear patterns (Figure 1), each consisting of a contiguous array of peptidoid components with molecular mass values ranging from ∼5 kDa to ∼60 kDa. Such a diffuse distribution pattern indicates a significant hydrothermal degradation of the native polypeptides during autoclaving. When compared to the electrophoretogram of PS, the CS displayed a slightly more limited distribution, but this might be due to a quantitative concentration of the components in PS due to removal through dialysis of the low molecular mass components. The other material evaluated in this study was an extract in methanol of the CS powder (CS-E), which was concentrated to dryness and then re-dissolved in an aqueous medium. The yield of extract was 5.34% relative to the weight of fraction CS and 0.83% relative to the total weight of cocoon material. Analysis carried out by using the BCA assay indicated the presence of 29% proteinaceous matter in this extract.

**Figure 2:**
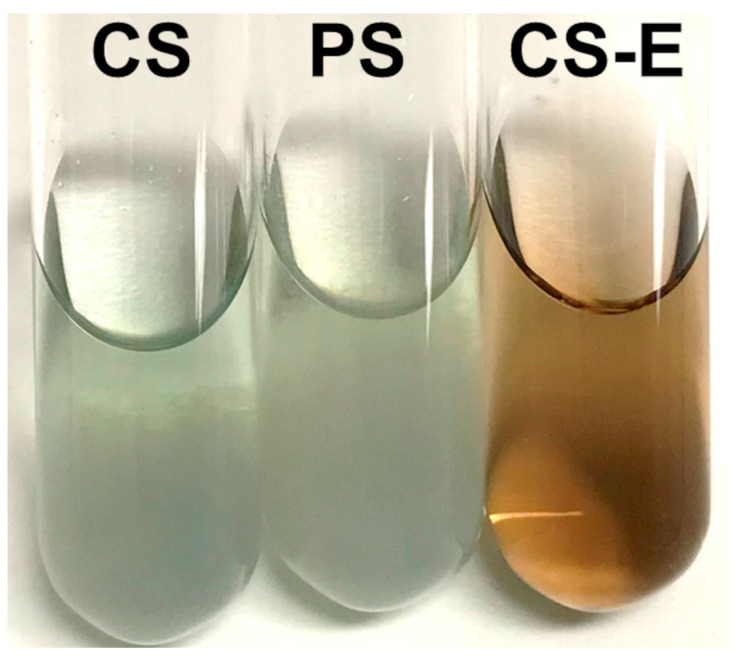
Staining of aqueous solutions of crude sericin (CS), purified sericin (PS), and extract of crude sericin (CS-E) following treatment with the DAC reagent in methanolic acidic medium. Photographs were taken after 1 h at room temperature. The red-brown coloration denotes the presence of flavonoids in the sample.

**Figure 3:**
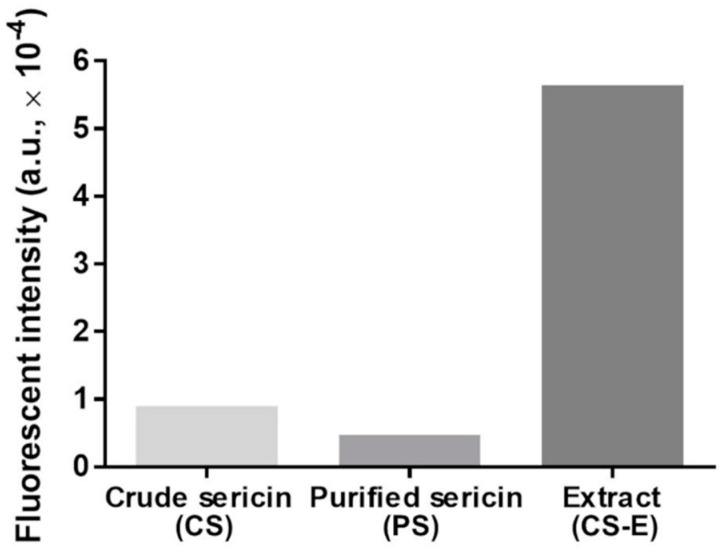
The fluorescence intensities of aqueous solutions of crude sericin (CS), purified sericin (PS), and extract of crude sericin (CS-E) following treatment with the 2-APB reagent, irradiation with 360-nm wavelength (excitation), and recording the emission spectra at 415–425 nm. The higher fluorescence intensity indicates the presence of flavonoids. Each bar is the result of one measurement.

To assess qualitatively and semi-quantitatively the presence of flavonoids, we employed two chromogenic methods. By reacting flavonoids with DAC, a deep red color shall develop [91]. The results of this test are shown in Figure 2, where such coloration is seen only in CS-E. In the other sericin fractions, a yellow-green coloration appeared along with precipitated matter that likely consists of sericinoid peptides. To bring additional proof of the presence of flavonoids in CS-E, a fluorescence method was applied based on the complexation reaction between flavonoids and 2-APB [92,93,94,95,96,97]. The resulting fluorescein measurements clearly indicated the presence of a substantial amount of flavonoids in CS-E as compared to CS or PS (Figure 3).

**Figure 4:**
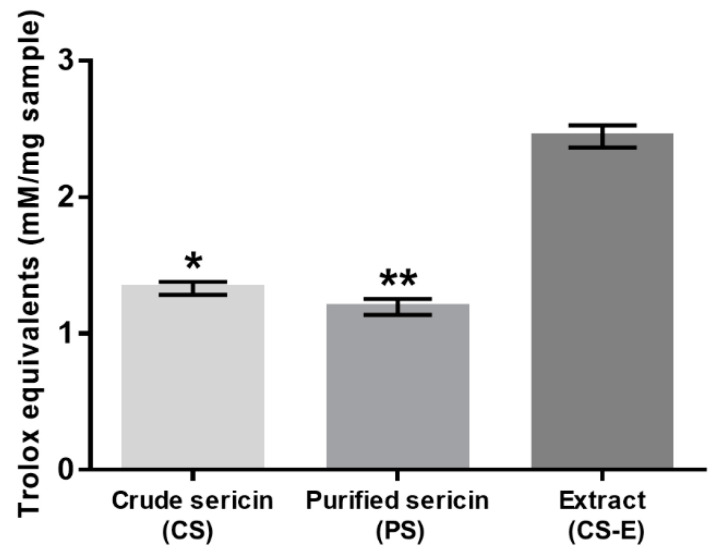
Antioxidant capacity of non-dialyzed (crude, CS) and dialyzed (purified, PS) sericin and of methanolic extract from crude sericin (CS-E) as determined by TEAC (ABTS^+·^) assay and expressed in Trolox equivalents. Bars represent mean values ± s. d. for *n* = 10. Statistical analysis was performed using the non-parametric Friedman test with Dunn’s multiple comparisons test. * *p* < 0.05; ** *p* < 0.0001. The difference between crude and purified sericins is not statistically significant (*p* > 0.2).

All three samples were evaluated with the TEAC assay to compare their intrinsic antioxidant capacity, and the results are shown in Figure 4. The sample CS-E displayed significantly higher antioxidant activity when compared to CS and PS, the latter two showing virtually the same levels of activity.

**Figure 5:**
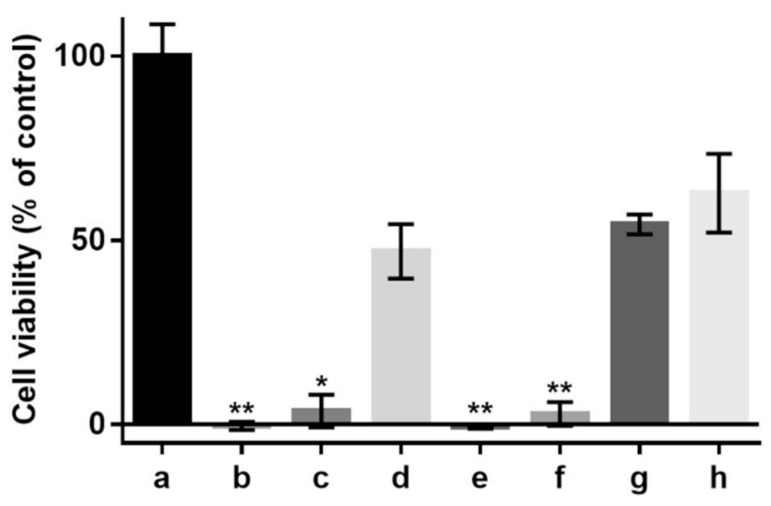
Effect of supplementation with sericin fractions on viability of 661 W murine retinal photoreceptor cells cultured in DMEM/10% FBS, without or in the presence of hydrogen peroxide (H202, 1.2 mM). (**a**) Control (no sericin, no oxidant); (**b**) control (no sericin, +oxidant); (**c**) CS, 1 mg/mL (+oxidant); (**d**) CS, 5 mg/mL (+oxidant); (**e**) PS, 1 mg/mL (+oxidant); (**f**) PS, 5 mg/mL (+oxidant); (**g**) CS-E, 1 mg/mL (+oxidant); (**h**) CS-E, 5 mg/mL (+oxidant). Bars represent mean values ± s. d. for *n* = 6. Statistical analysis was performed using the non-parametric Friedman test with Dunn’s multiple comparisons test. * *p* < 0.02; ** *p* < 0.002. The differences between cell viabilities in the control culture (**a**) and test culture samples (**d**,**g**,**h**) are not statistically significant (*p* > 0.2).

In the cell cultures investigated in this study, the antioxidant effects of various sericin fractions were found to be dose-dependent (Figure 5). While CS-E displayed statistically significant antioxidant activity at any concentration and CS showed such activity only at the higher concentration, PS did provide negligible protection, if any, to the cells against oxidative death.

**Figure 6:**
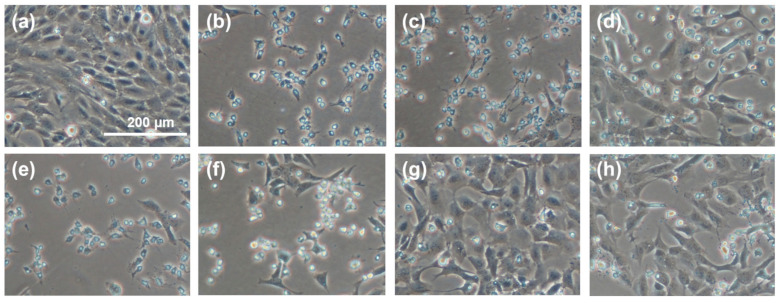
Micrographs of randomly selected cell growth areas illustrating the effect of supplemental sericin fractions on the proliferation and morphology of 661 W murine retinal photoreceptor cells under oxidative stress induced by hydrogen peroxide (H202, 1.2 mM by total media volume). (**a**) Control (no sericin, no oxidant); (**b**) control (no sericin, +oxidant); (**c**) CS, 1 mg/mL (+oxidant); (**d**) CS, 5 mg/mL (+oxidant); (**e**) PS, 1 mg/mL (+oxidant); (**f**) PS, 5 mg/mL (+oxidant); (**g**) CS-E, 1 mg/mL (+oxidant); (**h**) CS-E, 5 mg/mL (+oxidant). The scale bar in (**a**) is the same for all images.

The representative images in Figure 6 show a regional comparison between normally proliferating cells (Figure 6a), cells grown in the presence of an oxidant (H_2_O_2_) without any sericin fraction as a protective antioxidant (Figure 6b), cells grown in the presence of H_2_O_2_ in a medium supplemented with CS (Figure 6c,d) or PS (Figure 6e,f), and cells grown in the presence of H_2_O_2_ in a medium supplemented with CS-E (Figure 6g,h). The superior protective effect of fraction CS-E is convincingly reflected in the cellular morphology and growth patterns.

## 4. Discussion

The antioxidant capacity of BMSS may be regarded as a vestigial evolutionary feature contributing to the protective activities that the silk cocoon must perform. First, the cocoons, which are immobile and non-metabolizing entities, must be protected from infection and ensuing decay. This explains the presence in their composition of defense antimicrobial proteins [98,99,100,101]. Second, the cocoons are themselves responsible for the survival of the species by protecting the pupae from pathogens, predators, sunlight, humidity, and heat. A variety of proteins and non-protein substances in the silk thread composition fulfill such tasks. For instance, sericin in the *B. mori* cocoon material was found to be responsible for absorbing UV-A radiation, such as protecting the cocoon structure from oxidative damage during the pupal stage of development [102]. It is, therefore, to be expected that certain substances contained in the silk cocoon material will display identical or similar protective properties, including antioxidative capacity. 

The composition of non-protein matter in *B. mori* silk cocoon is traditionally reported [103,104] as 1.2–1.6% carbohydrates, ∼0.7% inorganic matter, 0.4–0.8% wax matter, and ∼0.2% pigments. The rest consists of the two major proteins in the silk thread, fibroin, and sericin, which are accompanied by much lower amounts of other proteins having either identifiable defense functions (enzymes, seroins, protease inhibitors) or other roles yet to be determined [99,100,101,104]. Regarding BMSS itself, the number and the distribution of constituting peptidoid units are still disputed. Between 2 and 15 polypeptides have been reported in the literature over the past century [6]. It has been also suggested [105] that the presence of many peptidoid fractions in BMSS is, in fact, an artifact due to hydrolytic degradative processes during isolation procedures. Closer to our times, however, genomic analysis has shown that BMSS must contain at least six major native polypeptides that are all distinctly biosynthesized in the middle gland of silkworms [106,107,108,109,110]. The molecular mass distribution of polypeptides in regenerated BMSS has been widely reported between 20 and 400 kDa [6].

The secondary metabolites in the plants that are eaten by silkworms are the main source of the non-protein substances in the cocoons they have spun. These metabolites are produced by plants without being directly involved in their own normal development, but they have essential protective and defensive roles that contribute to enhanced survival and reproduction for the host plant. All species that feed on plants, from insects to humans, use indirectly the secondary metabolites contained in the vegetable matter [111]. The range of secondary metabolites produced by plants is vast and includes terpenoids, carotenoids, phenolics (e.g., flavonoids, tannins), plant steroids (e.g., sterols), alkaloids (e.g., caffeine, nicotine), carbohydrates (e.g., saponins), and hydrocarbons. When the silkworms consume leaves, some of these phytochemicals can be selectively sequestered to the silk cocoon material, where they remain either as such [104,112,113] or as derivatives resulting from biosynthetic modifications occurring within the larvae’s gut [114,115,116]. Phytochemical sequestration is a highly selective process, as illustrated by the significant differences in the nature of non-protein components throughout the silkworm species. For instance, saponins and steroids have been identified in the silk cocoons produced by *Antheraea mylitta* silkworms but not in those produced by *B. mori* silkworms. In addition, the latter do not contain tannins or terpenoids. 

There is a considerable diversity of metabolites detected in the *B. mori* silk cocoons. No less than 45 metabolites have been identified in the wild *B. mandarina* cocoons, 28 of them being also present in the cocoons of its domesticated version, *B. mori* [104]. The following non-protein metabolites were found so far in the *B. mori* silk cocoons [104,112,113]: fatty acids and other carboxylic acids; carotenoids; flavonoids and other phenolics; carbohydrates and derivatives; amines; amino acids; urea and derivatives; hydrocarbons; and other organic compounds (e.g., hexanal, isopropanol, and glycerol). It is believed that many metabolites have defined roles in fulfilling the tasks required for protecting the larvae. For instance, flavonoids can shield the cocoons from UV-induced damage [114,117] and can enhance their resistance against oxidative damage [73,118]. 

In the present study, three types of samples were prepared from *B. mori* silk cocoons and were investigated in relation to their antioxidant capacity: crude BMSS (CS, non-dialyzed), purified BMSS (PS, dialyzed), and a methanolic extract of the crude sericin fraction (CS-E). To assure the solubility of BMSS fractions in aqueous media, the autoclaving stage was run for at least 4 h leading to the molecular mass distribution shown in Figure 1. Although shorter durations in the autoclave or employing procedures carried out at lower temperatures can lead to advanced preservation of the higher molecular mass components [90], such resulting sericin fractions are insoluble or only partially soluble in water, therefore unsuitable for evaluation in cell culture media. Regarding the extract (CS-E), it must be devoid of methanol and be soluble in an aqueous medium to enable its evaluation as an antioxidant. Therefore, methanol was removed by gentle evaporation to dryness, and the resulting residue was re-dissolved in water (for TEAC assay) or in DMEM (for cell culture). The bicinchonic acid (BCA) assay showed that the extract contained 29% proteinaceous matter that likely would include oligopeptides and amino acids that were dissolved and retained in the methanolic phase. This confirms the results of a previous study [60], where the ethanolic extracts of the crude sericin in five different strains of *B. mori* cocoons contained between 20 and 32% non-precipitable amino acids and low molecular mass peptides, the rest being non-sericin components such as flavonoids and other unidentified compounds.

TEAC assay indicated (Figure 4) that the antioxidative capacity of the extracted non-sericin components in CS-E was significantly higher than that of the water-soluble BMSS powder samples (CS and PS), the latter two showing almost the same levels of activity. While the identification of the individual chemical compounds in the CS-E was beyond the purpose of this study, it can be assumed that the non-sericin fractions include mostly flavonoids, as reported previously [60,65,71,72,73], substances known for their remarkable antioxidative properties. Flavonoids are indeed a major category of secondary metabolites in the mulberry leaves; for instance, 17 flavonoids have been identified in Korean mulberry leaves [119]. To investigate the status of flavonoids in CS, PS, and CS-E, we used two chromogenic analytical methods, which proved the presence of flavonoids, and that they exist predominantly in CS-E (see Figure 2 and Figure 3). As the contribution of flavonoids to the antioxidant properties of sericin formulations is a tenet of this study, we employed two different methods for the qualitative and semi-quantitative analysis of the samples in order to raise the level of confidence for proving the presence of flavonoids and their prevalence in CS-E. CS contains chaperone non-sericin substances that, after extraction, are retrieved within CS-E, while PS is supposed to contain only traces of them, if any. 

We cannot assert whether, or to what extent, the peptidoid components co-extracted in methanol have contributed to the total antioxidant activity of the CS-E fraction. However, by examining the antioxidant activities in cell culture (Figure 5), it becomes evident that the peptidoid substances in CS-E may not have any contribution and that the activity displayed by CS at higher concentration (Figure 5d) is likely due to the presence of non-sericin substances that would be eventually transferred to CS-E by solvent extraction. The purified sericin, PS, where such substances were removed by the process of dialysis, did not display antioxidant properties in cell culture (Figure 5e,f). Our previous results [78], where insoluble gels containing sericin were used as solid substrates for cell growth, have shown no antioxidative protection for the cells suggesting that pure sericin is not an antioxidant per se. On another note, the fact that PS fraction has shown, by the TEAC assay (Figure 4), a level of antioxidant activity similar to that of CS might be attributed to the difference between a chemical test and an evaluation in cell culture. The TEAC assay reflects straightforward the ability of a substance to participate in a specific chemical reaction, while the assessment in cell culture of the same substance is an intricate process influenced by factors such as number of cells, amounts of antioxidant or oxidant, and differences in the reaction mechanisms involving the antioxidant. Therefore, a direct correspondence between the two assays is rather incongruous. As a related recommendation [32], a substance shall not be called an antioxidant at cellular level or in vivo only because any of the available chemical assays has revealed antioxidant properties. 

The choice of a cell line in our study was purposeful: the photoreceptor cells (cones and rods) are crucially involved in the pathophysiology of age-related macular degeneration (AMD), currently the major cause of visual impairment and blindness in the Western world [120]. AMD is a complicated neurodegenerative process affecting the aging retina and leading to progressive visual loss and irreversible blindness. The currently available therapies are limited and effective only in certain categories of patients. Although the pathomechanisms leading to AMD are not fully elucidated, it is believed that oxidative stress, associated with an excessive generation of ROS, peroxidative processes, and chronic inflammation, is the significant factor triggering damage to cells that results in dysfunction of the retina in AMD and in other retinal degenerations [120,121,122,123,124]. The retinal photoreceptor cells, primarily the cones, are specifically amenable to oxidative damage as they are nonproliferative cells that lack detection systems for ROS-induced damage to nucleic acids at the checkpoints in the cell cycle. They have a high demand for oxygen and are under continuous exposure to light. 

Discovering antioxidants that can be placed in the subretinal space, with an aim to reduce oxidative stress and protect the sensitive photoreceptor cells, is pivotal to potential treatments for AMD and other blinding diseases. However, more extensive knowledge of their in vivo mechanism of action, absorption, modification, distribution, and real benefits is imperative. While evaluation in single-cell culture systems is essential to this aim, a lengthy and laborious process is still needed in order to achieve clinically useful antioxidants.

## 5. Conclusions

Certain sericin fractions isolated from *B. mori* silk cocoons possess antioxidative capacity, as demonstrated in the present study by a photochemical assay and in cultures of murine retinal photoreceptor cells. While the crude sericin shows some activity at higher concentration, the highest level of antioxidant activity is displayed at cellular level by the sericin-associated non-peptidoid components extracted in a solvent from crude sericin, where the presence of flavonoids can be demonstrated chromogenically. The purified sericin fraction shows negligible activity, if any. Antioxidants able to protect the photoreceptor cells are relevant for the development of therapeutic strategies against age-related macular degeneration (AMD) and other eye pathologic conditions associated with oxidative stress. The extracts of sericin are promising natural antioxidants that may be effective in an in vivo pathologic situation.

## Data Availability

The authors can confirm that all relevant data are included in this published article. The authors can provide upon request samples of the materials described in the article.

## Sample Availability

Samples of the compounds are not available from the authors.

## Abbreviations

2-APB	2-Aminoethyl diphenylborinate
AMD	Age-related macular degeneration
BMSS	*Bombyx mori* silk sericin
CS	Crude sericin
CS-E	Methanolic extract from crude sericin
DAC	4-(Dimethylamino)cinnamaldehyde
DMEM	Dulbecco modified Eagle’s medium
FBS	Fetal bovine serum
HBSS	Hank’s balanced salt solution
MMCO	Molecular mass cut-off
MTT	3-(4,5-Dimethylthiazol-2-yl)-2,5-diphenyltetrazolium bromide
PS	Purified sericin
ROS	Reactive oxygen species
SDS-PAGE	Sodium dodecyl sulphate-polyacrylamide gel electrophoresis
TEAC	Trolox equivalent antioxidant capacity
